# A Mirror Bilateral Neuro-Rehabilitation Robot System with the sEMG-Based Real-Time Patient Active Participant Assessment

**DOI:** 10.3390/life11121290

**Published:** 2021-11-24

**Authors:** Ziyi Yang, Shuxiang Guo, Hideyuki Hirata, Masahiko Kawanishi

**Affiliations:** 1 Graduate School of Engineering, Kagawa University, Takamatsu 761-0396, Japan; s21d502@kagawa-u.ac.jp; 2 Key Laboratory of Convergence Medical Engineering System and Healthcare Technology, The Ministry of Industry and Information Technology, Beijing Institute of Technology, Beijing 100081, China; 3 Department of Intelligent Mechanical Systems Engineering, Kagawa University, Takamatsu 761-0396, Japan; hirata@kagawa-u.ac.jp; 4 Department of Neurological Surgery, Faculty of Medicine, Kagawa University, Takamatsu 761-0793, Japan; mk@kms.ac.jp

**Keywords:** bilateral rehabilitation, exoskeleton robotics system, surface electromyography (sEMG), isometric force estimation, upper limb elbow joint rehabilitation

## Abstract

In this paper, a novel mirror visual feedback-based (MVF) bilateral neurorehabilitation system with surface electromyography (sEMG)-based patient active force assessment was proposed for upper limb motor recovery and improvement of limb inter-coordination. A mirror visual feedback-based human–robot interface was designed to facilitate the bilateral isometric force output training task. To achieve patient active participant assessment, an sEMG signals-based elbow joint isometric force estimation method was implemented into the proposed system for real-time affected side force assessment and participation evaluation. To assist the affected side limb efficiently and precisely, a mirror bilateral control framework was presented for bilateral limb coordination. Preliminary experiments were conducted to evaluate the estimation accuracy of force estimation method and force tracking accuracy of system performance. The experimental results show the proposed force estimation method can efficiently calculate the elbow joint force in real-time, and the affected side limb of patients can be assisted to track output force of the non-paretic side limb for better limb coordination by the proposed bilateral rehabilitation system.

## 1. Introduction

Hemiplegia, which is a common sequela after post unilateral stroke, always refers to the hemiparesis on the contralateral side of the upper limbs leading to disability on one side [1]. Due to the asymmetrical motor function between bilateral side limbs, the impaired paretic arms can disorder the bimanual coordination function and disrupt the inter-hemispheric balance, which reveals the interlimb coordination after stroke may be a crucial point for stroke motor rehabilitation [2]. To address this kind of spatiotemporal incoordination of bilateral side limbs, bilateral rehabilitation training is considered as a promising way for hemiplegic recovery, which can activate the ipsilesional primary motor area (M1), supplementary motor area (SMA), and primary sensory cortex (S1) as well as enhance the intra-hemispheric and inter-hemispheric connectivity within the sensorimotor network and the cortical motor system. Bilateral rehabilitation training is more effective than unilateral arm training [3]. On the other hand, mirror visual feedback (MVF), a kind of mirror therapy of neurorehabilitation for hemiplegia [4], was proven that it can efficiently induce the human primary motor cortex (M1) for motor function recovery [5]. This phenomenon might be utilized to accelerate motor control rehabilitation processing.

Due to a lack of medical sources and the increasing number of stroke patients, robot-aided rehabilitation is proposed to accelerate recovery processing based on the aforementioned neurological principle [6,7]. For hemiplegic patients, the bilateral rehabilitation robotics system is designed with a special feature that can allow patients to perform the symmetric movements of the paretic side limb using the motor information of the non-paretic side limb. Leonardis et al. [8] developed the BRAVO hand exoskeleton for rehabilitation, which can assist the paretic side limb to grasp the real object for bilateral training. Gasser et al. [9] designed an upper limber exoskeleton for daily life activities assistant for patients with hemiparesis. Miao et al. [10] presented a platform robotics system with a subject-specific workspace for bilateral rehabilitation training. In our previous works, a series of exoskeleton robotic systems have been proposed for bilateral rehabilitation in the last decade, including a three degree of freedoms (DOF) portable exoskeleton [11,12], and two kinds of compliance actuator-integrated exoskeleton device [13,14].

Furthermore, the surface electromyography (sEMG) is a muscle drive signal that contains the motor information of the central nervous system (CNS) and brain, which is always utilized for rehabilitation assessment [15], human intention prediction [16,17,18], human movement classification [19,20,21], prosthesis control [22,23,24], and rehabilitation robot control [25,26,27]. As the reference [28], the neuroplasticity can be induced by the patient’s active participation that requires the rehabilitation training system should be implemented with an active participant assessment function. The real-time patient participation assessment not only can let therapists clearly know the training effect but also can encourage the patients to focus on the training task and improve the training effect. In the isometric bilateral lifting training, the output force is the key evaluation metrics for active participation and muscle state. Therefore, the active forces of the antagonistic muscle pairs of elbow joint should be evaluated for participation assessment.

The sEMG-based human active force estimation is discovered as it can intuitively reflect muscle motor unit action potentials (MUAPs) for active muscular force evaluation [29]. Zonnino et al. [30] proposed a muscular model-based isometric force estimator using sEMG signals. As the complexity of the muscular model and substantial calculation load of the muscular model parameter, the model-free force estimation method using machine learning is also widely used in rehabilitation scenarios [31,32]. In our previous works, we compared the estimation accuracy and calculation loads of these two methods in the isometric force estimation task [33]. The neural network-based method has the advantage of fast and convenience setup without a human body parameter setting, which is suitable for rehabilitation scenarios.

**Figure 1:**
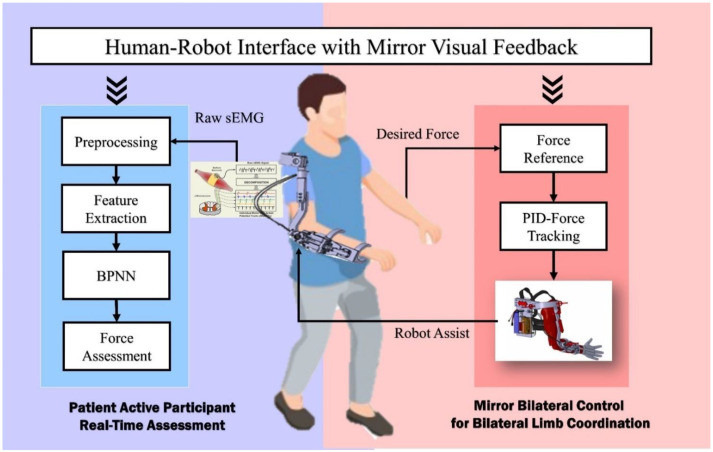
Conception diagram of the proposed mirror bilateral neuro–rehabilitation system with real–time sEMG–based patient active participant assessment.

As mentioned, a novel mirror bilateral neuro-rehabilitation system with sEMG-based real-time active force assessment is proposed in this paper and preliminarily tested for the upper limb elbow joint bilateral isometric force coordination. The conception diagram is shown in Figure 1. The patient can be allowed to perform the synchronic and isometric bilateral lifting task of the elbow joint by robotic assistance within a mirror visual feedback-based human–robot interface. During the training process, the patient active force of the paretic side limb can be estimated in real-time by the sEMG signals for patient active participation assessment.

The paper structure is organized as follows: Section 2 introduces the robotics system in mechanical structure and mirror visual feedback-based human–robot interface design. The sEMG-based isometric force estimation method is provided in detail for signal preprocessing, feature extraction, and neural networks preparation in Section 3. Then, we introduce the control framework for the real-time bilateral lifting task in Section 4. The experimental setup and preliminary results are provided and analyzed in Section 5. The discussion is based on the experimental results, which includes the comparison of the sEMG-based force estimation performance with the state-of-art and the effect of the MVF and robot-assistance performance. Finally, the conclusion and future work are drawn in Section 7.

## 2. Mirror Bilateral Neuro-Rehabilitation System Overall

In this section, a powered variable stiffness exoskeleton device (PVSED), as the hardware platform, is reviewed in mechanical design and the visual feedback-based human–robot interface is introduced for a mirror bilateral neuro-rehabilitation.

### 2.1. PVSED Hardware

**Figure 2:**
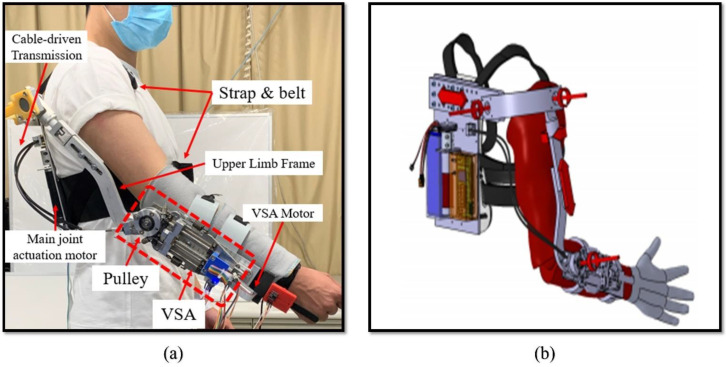
The mechanical design of the PVSED: (**a**) the prototype of the PVSED with a subject and (**b**) the adjustable and flexible structure of the PVSED.

In this study, the PVSED was utilized for aiding the subject to finish the mirror bilateral rehabilitation training tasks. The PVSED was developed in our previous research, which not only can assist flexion and extension motion of the upper limb elbow joint, but also can independently regulate the stiffness via a variable stiffness actuator (VSA). The detailed information of the PVSED was introduced as the reference [34]. For easier and clearer reading, the PVSED is reviewed in this section, shown in Figure 2a. There is one active degree of freedom on the elbow joint and five passive degrees of freedom on the PVSED. The PVSED consists of a back frame, a shoulder frame, and an upper limb frame. All of these frames were designed with an adjustable and flexible structure to adapt subject-specific body sizes in real rehabilitation scenarios shown in Figure 2b. There are two different actuators in the PVSED, including the main actuator system and an independent VSA as stiffness actuator system. In the main actuator system, a cable-driven transmission structure was selected for high back-drivability and lightweight load for the patients. A Maxon RE-30 Graphite Brushes Motor was implemented on the back broad, which is attached to the back of the patient by shoulder straps and body belts as the main actuator system for driving the cable transmission. On the other side of cable transmission, a pulley of the mainframe on the elbow joint was connected for rotation of flexion and extension motion of the elbow joint. The VSA was also integrated on the mainframe to regulate the real-time stiffness output. The output stiffness, as well as the force, was generated by the deviation between the mainframe and the output link coupled by a pair of antagonistic springs. In this study, only the high-stiffness condition was selected to assist simulation as the normal non-compliance rehabilitation robot.

### 2.2. Mirror Visual Feedback-Based Human-Robot Interface

**Figure 3:**
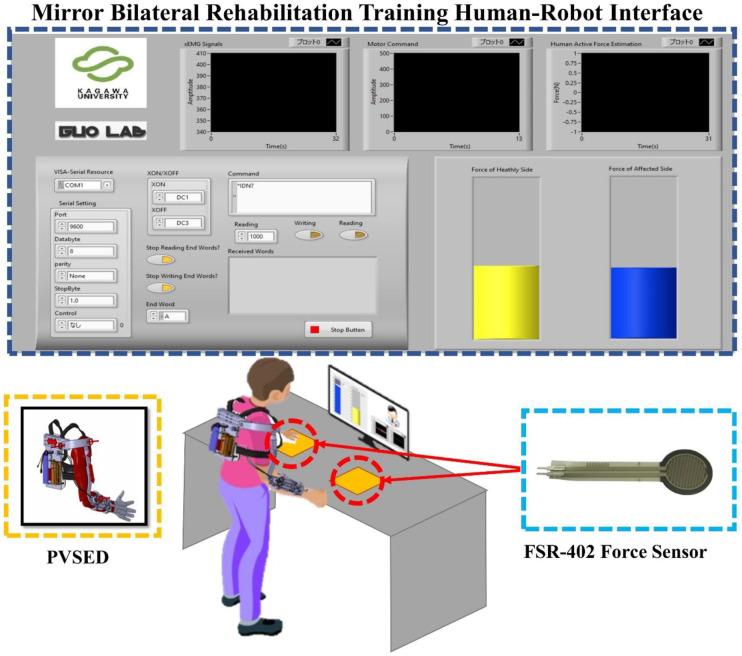
The system configuration of the mirror bilateral rehabilitation training. The navy–blue dotted box is the visual feedback–based human–robot interface. The yellow dotted box is the PVSED worn by the patient. The blue box is two thin-film force sensors symmetrically placed on the table.

Effective real-time visual feedback is crucial to enhance motor learning in physical and cognitive rehabilitation [35]. For improving the mirror bilateral rehabilitation task performance, a visual feedback-based human–robot interface was designed and implemented into the mirror bilateral neuro-rehabilitation system (developed within the LabVIEW, NI, Austin, TX, USA). The motivation of the visual feedback-based interface is to quantitatively evaluate the difference between the bilateral force and realize the intuitive feedback to the patients for a better training effect. For the mirror bilateral isometric force training task, the bilateral force signals are recorded by the thin-film force sensor (FSR-402, Interlink Electronics, Camarillo, Irvine, CA, USA) from both the non-paretic side and the affected side, and turned to the visual feedback through two symmetrical vertical scroll bar models in the interface in real-time. At the same time, the sEMG signals of the affected side are collected for active force estimation (introduced in Section 3). The whole system configuration and the visual feedback human–robot interface are shown in Figure 3.

### 2.3. The Mirror Bilateral Training Protocol of Upper Limb Elbow Joint

In this study, a mirror bilateral isometric force training with visual feedback has been designed for improving motor learning and regaining motor control skills in patients. There are three different phases in the training process, including the offline learning phase, online validation phase, and real-time assist phase. The process involves the subjects sitting on the chair comfortably and placing their forearms on the table, which ensures their hands naturally touch the force sensor. Two Ag/AgCl bipolar surface sEMG electrodes were placed on the biceps and triceps of both side limbs for sEMG signals collection through the Personal-EMG device (Oisaka Electronic Equipment Ltd., Fukuyama, Hiroshima, Japan). The bilateral force signals were then displayed by the two symmetric vertical bar models and the sEMG signals shown in graph models in the human–robot interface configuration. In the offline learning phase, the active isometric force estimation is established by a neural network method for muscular force assessment. The subject is instructed to perform an isometric force output against the force sensor by their healthy side. The force signals and sEMG signals are recorded in real-time for training the learning algorithm. After the learning algorithm training, the online validation phase begins for ensuring the efficiency and safety of the trained estimation model. The subject repeats the same motion in the online validation phase but the estimated force results are calculated by the trained model and shown on the screen in real-time. The poor performance estimation model is rejected and retrained for the safety consideration until the estimation performance is acceptable. The real-time assist phase is performed once the online validation phase is finished. As the one disability side of the hemiplegia patients, the trained force estimation model is unitized as the active force assessment of their affected side. The subject with the PVSED is instructed to perform the mirror bilateral motion with the equal force output against the force sensors to maintain the same height of the two symmetrical vertical scroll bar models. The bilateral isometric force information is recorded and the error between both sides is then calculated as the control input of the bilateral limb coordination controller (introduced in Section 4).

## 3. sEMG-Based Isometric Active Force Estimation

The sEMG-based active isometric force estimation method is introduced as the following three subparts for real-time muscle active force assessment.

### 3.1. sEMG Signal Processing

**Figure 4:**
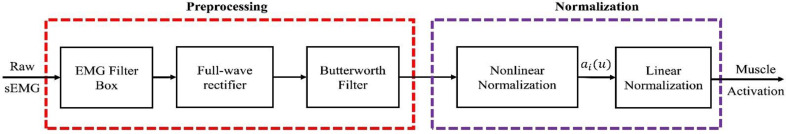
sEMG signals preprocessing and normalization for obtaining the muscle activation.

**Figure 5:**
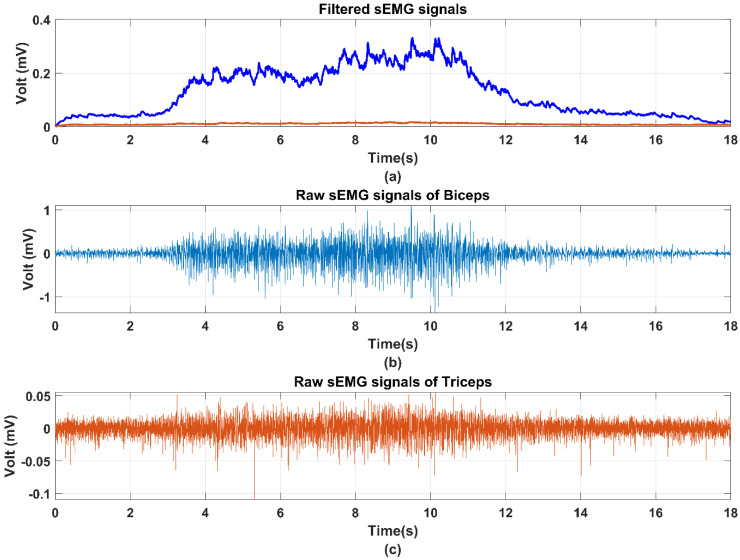
sEMG signals preprocessing and normalization for obtaining the muscle activation: (**a**) the blue line is the filtered sEMG signals of the biceps, and the orange line is the filtered sEMG signals of the triceps, (**b**) raw sEMG signals of biceps. (**c**) Raw sEMG signals of triceps.

Signal preprocessing is necessary for removing the noises and DC offset due to the instability of the sEMG signals. After the sEMG signals’ acquisition of 1000 Hz sampling rate (Section 2.3), the raw sEMG signals are processed by a Personal-EMG filter box (Oisaka Electronic Equipment Ltd., Fukuyama, Hiroshima, Japan) for removing the DC offset. Then, the filtered sEMG signals are rectified by a 50 Hz notch filer for full-wave rectification. The processed sEMG signals can be obtained after a four order Butterworth filter with 10–500 Hz cut frequency. Due to the individual difference and instability of sEMG signals, the normalization processing should be implemented after the Butterworth filter to obtain the normalized sEMG signals from 0 to 1 by maximum voluntary contraction (MVC). The completed signal processing is shown in Figure 4. The comparison results of the raw signals and filtered sEMG signals are shown in Figure 5 for clear observation.

### 3.2. Feature Extraction

**Figure 6:**
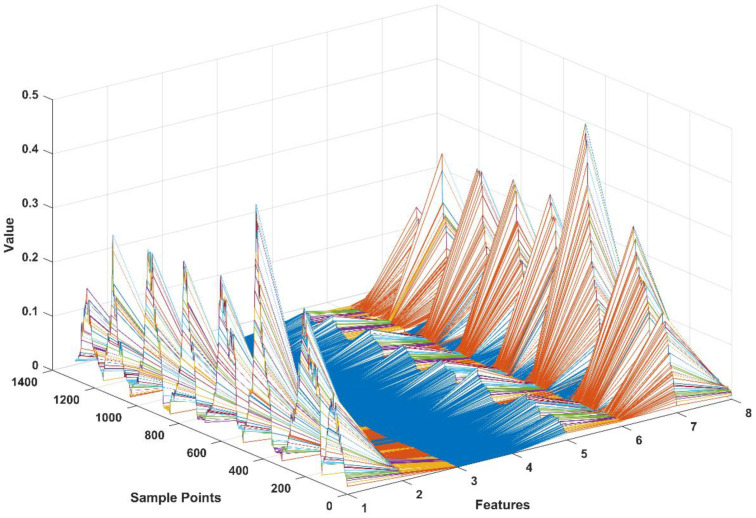
sEMG signals preprocessing and normalization for obtaining the muscle activation.

As proven in the previous study [19], the multi-features of sEMG signals with time-domain feature and frequency domain feature contain more efficient and internal information than a single feature. To obtain the accurate force estimation performance, a novel time-domain multi-feature set was selected and utilized as the input vector of the neural network. Here, we review these features and their descriptions in Table 1. The multi-feature vector consists of four time-domain features, including mean absolute value (MAV), root mean square (RMS), difference absolute standard deviation value (DASDV), and wavelength (WL). Each feature vector is calculated from one channel of sEMG signals of one muscle by a 0.2 s sliding window method in real-time, and the multi-feature vector space is shown as Figure 6.

### 3.3. BPNN

**Figure 7:**
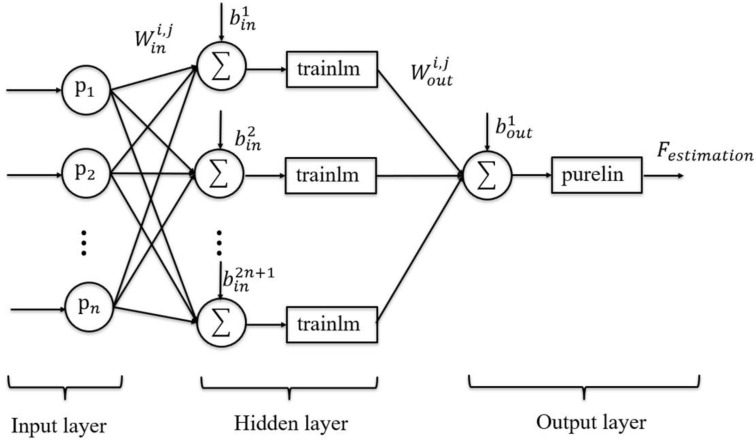
The structure of the BPNN model for human active force estimation and real-time force assessment.

As the nonlinear relationship between the sEMG signals and human motor joint output force, the backpropagation neural network is employed for estimating the human active motor joint output force. The BPNN structure in this study is designed with three layers containing: an input layer (X(n)), a hidden layer (h(n)), and an output layer (*Y*(n)), as shown in Figure 7. The input of the BPNN is the multi-feature vector calculated from the biceps and triceps. Because there are four features in one multi-feature vector, the number of input layer neurons totals eight. The number of hidden layer neurons is calculated by the equation:

(1) $$N_{Hidden}=\log_{2}(N_{Input})$$

where the $N_{Input}$ is the number of input layer neurons. The hidden layer neurons are set as three. The physical meaning of the output layer is the elbow joint output force. Therefore, the output layer only has one neuron representing the estimated force. The hidden layer can be represented as follows:

(2) $$h_{j}^{i}\left(n\right)=Sigmoid\left(\sum_{i=1}^{2}w_{in}x_{i}\left(n\right)-t\right)$$

where the $w_{in}$ is the weight value between the i-th input neuron and *j*-th hidden neuron. The t is a threshold of each hidden layer neuron to guarantee accuracy and convergence. The sigmoid function has been selected as the activation function in the hidden layer. For the output layer results, it can be calculated as the following equation:

(3) $$Y\left(n\right)=w_{out}\left[\frac{2}{1+e^{-2\left(\sum w_{in}X\left(n\right)-t\right)}}-1\right]+b_{out}$$

where the $w_{out}$ is the weight value between the *i*-th hidden neuron and the output layer neuron. The $b_{out}$ is a threshold of the output layer neuron. For BPNN model training, the 70% sEMG-force data set collected in the offline training phase is utilized and the other 30% is used for model validation. The output of the BPNN model should be anti-normalized to get the estimated force results. The trained and validated BPNN model is verified by the online validation phase and the high-performance BPNN model is used for real-time force estimation.

## 4. Mirror Bilateral Control for Bilateral Limb Coordination

The mirror bilateral rehabilitation training aims to assist the affected side limb to finish the mirror motion following the non-paretic side motion guidance. In the bilateral rehabilitation robotics system, a special characteristic is that the motion information of the non-paretic limb is delivered to the affected side limb within mirror-symmetric guidance as the most suitable training for the patients themselves. The hemiplegia patients can regain motor control skills and more importantly, the bilateral limb coordination to improve the motor cognition of the bilateral brain hemisphere.

In this study, a mirror bilateral isometric force training with visual feedback is designed for the high injured patients or initial stage of rehabilitation as the isometric force output is the easiest movement task of rehabilitation. To assist the patient appropriately, the output force error was calculated as the assist metric.

(4) $$F_{error}=F_{health}-F_{affected}$$

**Figure 8:**
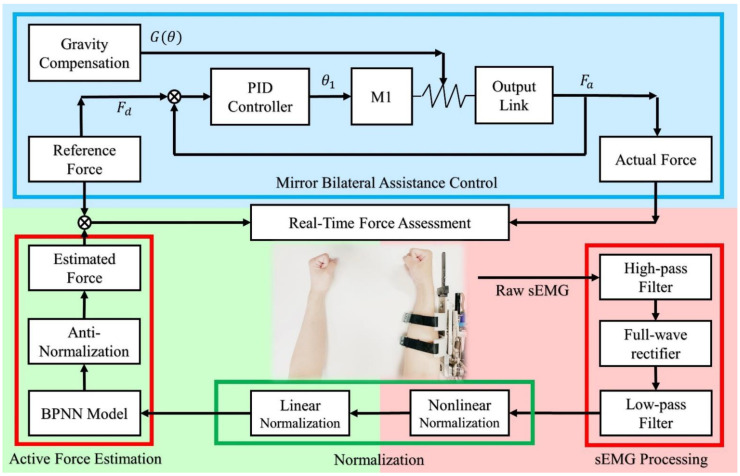
Control frameworks of the robot-aided mirror bilateral isometric force rehabilitation training. There are three main parts in the whole system including Active force estimation for real-time assessment, sEMG processing, and mirror bilateral assistance control for real-time robot-aided rehabilitation training.

For the precise assistant force control of the PVSED, the dynamics of the active DOF of the elbow joint should be considered as follows [36]:

(5) $$J_{m}\ddot{\theta_{j}}+B_{m}\dot{\theta_{j}}+G\left(\theta_{j}\right)=\tau_{VSA}+\tau_{human}$$

(6) $$J_{1}\ddot{\theta_{1}}+B_{1}\dot{\theta_{1}}+\left(\tau_{j}+\tau_{human}\right)/\gamma =T_{m1}\;$$

(7) $$T_{m1}=k_{m1}i_{m1}$$

(8) $$\tau_{VSA}=K\left(\theta_{2}\right)\cdot \left(\theta_{j}-\theta_{1}\right)$$

where the $\theta_{j}$, $\dot{\theta_{j}}$, and $\ddot{\theta_{j}}$ represent the angle, angular velocity, and angular acceleration of the output link. Similarly, the $\theta_{1}$, $\dot{\theta_{1}}$, and $\ddot{\theta_{1}}$ are the angle, angular velocity, and angular acceleration of the mainframe. The $J_{m}$ and $B_{m}$ are the inertia moment of the motor rotor and the damping coefficient of the output link, respectively. The $J_{1}$ and $B_{1}$ are the inertia moment of the motor rotor and the damping coefficient of the mainframe respectively. The $G\left(\theta_{j}\right)$ denotes the gravity of the human forearm and the PVSED. The $\tau_{VSA}$ represents the output torque of the VSA and it can be obtained by Equation (8) related to the stiffness and deviation angle. The parameter γ is the torque transmission ratio of the main actuator system, which is driven by the motor m1 with the motor torque constant $k_{m1}$ and its’ torque $T_{m1}$ is controlled by the motor current $i_{m1}$. Due to the isometric force constraint, the angular can be considered as a constant so that the angular velocity and angular acceleration can be ignored. As mentioned, the only high stiffness condition of the PVSED is discussed in this paper, which means the robotic stiffness is 118.49 Nm/rad. For precise and rapid force tracking performance, a PID controller was employed in the control system. The input is the force of the healthy side and the output is the motor current. The force of the affected side would be feedback to the input for feedback control. It should be noted that although the angular velocity and angular acceleration can be ignored in the isometric force output task, the gravity of the PVSED and human forearm should be compensated to set an initial position of forearms for comfortability and precision. The overall control framework is shown in Figure 8.

## 5. Experimental Setup and Results

### 5.1. Experimental Setup

There are two healthy subjects with no muscular disorder history (males, age: 25 and 22 years old; weight: 69 kg and 59 kg, height: 177 cm and 174 cm) involved in this study for mirror bilateral isometric force training. The PVSED was fixed to a metal structure and adjusted to fit each subject-specific height. Then, the sEMG electrodes will be placed on the subject’s arm for real-time sEMG collection and MVC test. The location of electrodes corresponding to the triceps was at 50% on the line between the posterior crista of the acromion and the olecranon at two-finger-width medial to the line.

**Figure 9:**
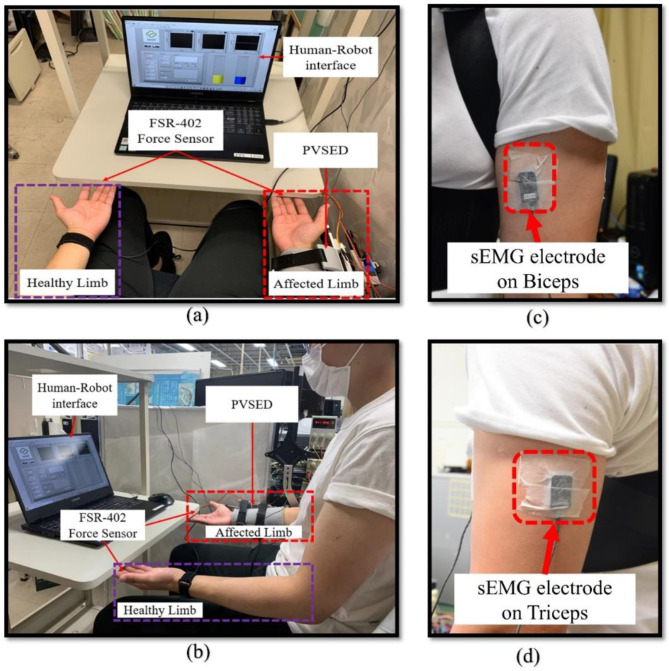
Experimental setup: (**a**) front view of the experimental setup, (**b**) lateral view of the experimental setup, (**c**) sEMG electrode location of biceps, (**d**) sEMG electrode location of triceps.

The reference electrodes were placed on the styloid process of the ulna of the wrist joint. The experimental setup is shown in Figure 9. In the experimental trials, the subject was instructed to perform the equal force of the bilateral side limbs on the force sensors placed under the experiment platform. When their max active effort was reached, they were asked to relax their arms. There are a total of five trials for each subject in the offline training phase. The same task is performed after the learning model training for the online validation phase. Finally, the real-time assist phase can be carried out after the well-performance model is selected. All experiments were conducted within the experimental requirements of the Institutional Review Board (IRB) in the Faculty of Engineering Kagawa University (Ref. No. 01-011).

### 5.2. Experimental Results

**Figure 10:**
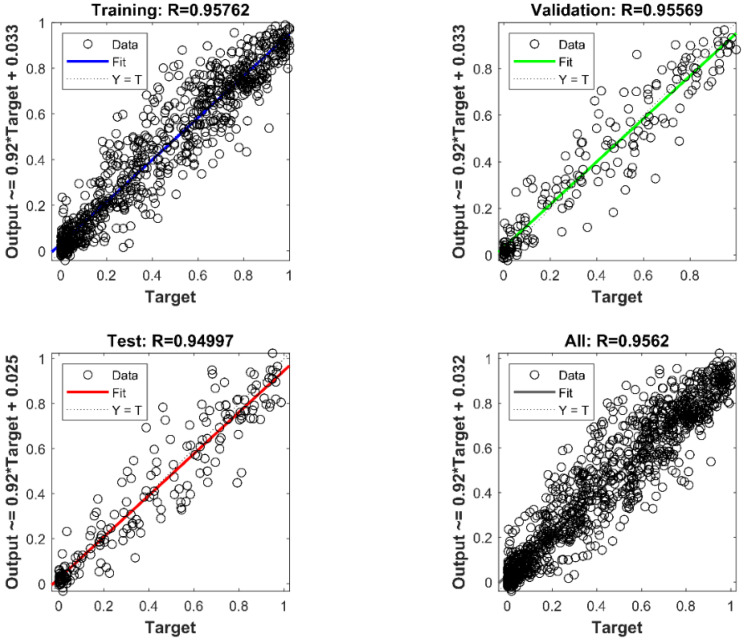
Regression results of BPNN model training, validation, testing, and overall performance.

**Figure 11:**
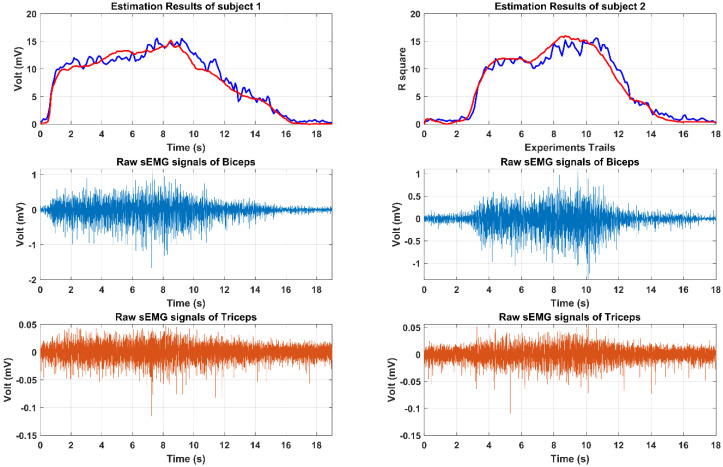
The real–time active force estimation results by sEMG signals from biceps and triceps. The left graph shows the results of the subject 1 and the right graph is subject 2.

**Figure 12:**
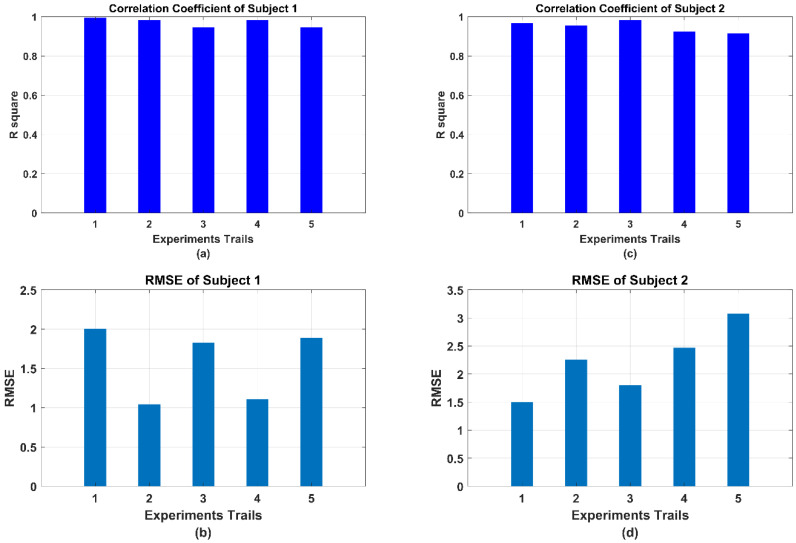
The evaluation results of the sEMG–based active force estimation method by correlation coefficient and RMSE: (**a**) correlation coefficient results of subject 1, (**b**) RMSE results of subject 1, (**c**) correlation coefficient results of subject 2, and (**d**) RMSE results of subject 2.

(1) Estimation Performance: For sEMG-based force estimation, the BPNN model should be set up and trained at first. Each subject was instructed to perform the isometric force output process from the relaxing state to the max voluntary output state five times. During the subject increasing the output force of the bilateral limb, the sEMG signals were accordingly increasing from the 0.2 mV of the relaxing state to the 0.6 mV. The BPNN model training results are shown in Figure 10, where the linear regression results of the model training, model validation, model test, and total performance are 0.95762, 0.95569, 0.94997, and 0.9562, respectively. To clearly observe the real-time estimation performance, a real-time estimation result of the experimental trails is shown in Figure 11. From Figure 11, even if the output force was not linear, the estimation result could also track this nonlinear trend by sEMG signals in real-time. In the end, the correlation coefficient and root mean square error (RMSE) results of all 10 times of the experimental trials have been calculated as the following equations, shown in Figure 12.

(9) $$\mathrm{RMSE}=\sqrt{\frac{1}{n}\overset{N}{\underset{n=1}{\sum }}{\left(F_{E}-F_{A}\right)}^{2}}$$

(10) $$R^{2}=\left(\frac{\sum F_{E}F_{A}-\frac{\sum F_{E}F_{A}}{N}}{\sqrt{\left(\sum F_{E}-\frac{{\left(F_{E}\right)}^{2}}{N}\right)\left(\sum F_{A}{}^{2}-\frac{{\left(\sum F_{A}\right)}^{2}}{N}\right)}}\right){\;}^{2}$$

where the $F_{E}$ denotes the estimated force and $F_{A}$ represents the actual force. The parameter N is the number of sample points. All the calculations and data analysis were processed by MATLAB (MathWorks, MA). The max RMSE is under 3.5 N and the min RMSE is over 1 N. Similarly, the highest correlation coefficient is 99.29, and the lowest one is 91.33. It is noted that the effect of the triceps is not obvious during the force output process. This phenomenon was possibly caused by the manner of output force against the force sensor. In this study, the bilateral output force was designed as “lifting force of the elbow joint” which refers to the flexion movement of the elbow joint. As the biomechanics, this flexion movement is mainly driven by the contraction of the biceps, and the triceps are in the extension state during this movement. On the contrary, if the manner of output force is designed as the “pushing down of the elbow joint”, it can be predicted that the triceps will take the domination effect rather than the biceps. However, considering the synergy influence of the wrist joint in the “pushing down movements”, the “lifting movement” was selected for preliminary evaluation. Both lifting and pushing down movements will be considered as the future works of this mirror bilateral neuro-rehabilitation system.

**Figure 13:**
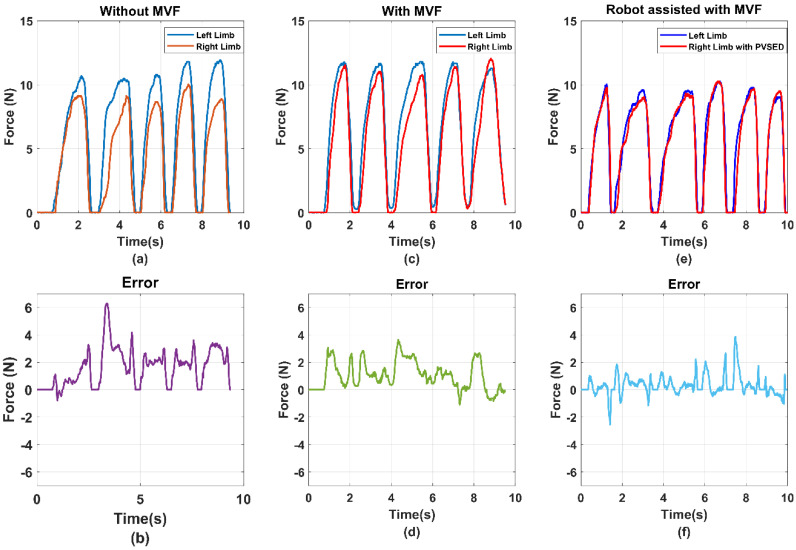
The comparison results of subject 1 in the three conditions, with/without the MVF and robotic assistance with MVF. (**a**) the bilateral isometric force task without MVF; (**b**) the errors of the bilateral isometric force task without MVF (**c**) the bilateral isometric force task with MVF (**d**) the errors of the bilateral isometric force task with MVF (**e**) the bilateral isometric force task with MVF and Robotics assistance. (**f**) the errors of the bilateral isometric force task with MVF and Robotics assistance.

(2) Real-time assistant performance: For evaluating the robot assist effect in the real-time assist phase, the output force signals of both side limbs have been compared in Figure 13. Due to the bilateral rehabilitation training requirements that use the information of healthy side limb to guide the affected-side limb, the healthy side output force was set as the reference signals and the robot assisted the affected side limb in tracking the reference force. As the implementation of the PID force tracking controller, the affected side output force was almost the same as the reference, which can be observed in Figure 13. The error signal between the reference force of the non-paretic side and robot-aided force of affected force was also shown, which was utilized as the input signals of the PID controller.

Considering the safety of the human–robot interactions, the parameter of the PID controller was set as relatively low, which may reduce the force tracking performance so that the bilateral output error could be over 5 N, as shown in Figure 13b. The other reason is that the high-stiffness condition of the PVSED was selected in this study. For the rehabilitation scenario, the high stiffness of the robotics may lead to the high intensity and high interaction force for motor skill learning and regaining or motor control skills. However, it will also increase the potential risk of secondary injury due to the strong interactions. Therefore, the force tracking performance was compromised for ensuring the training safety. In future works, the low-stiffness output condition can be explored for high compliance human–robot interaction.

## 6. Discussion

This study’s main purpose is to mirror a bilateral neuro-rehabilitation robotics system with sEMG-based patient active participant assessment, which uses the mirror visual feedback and robot assistant to induce bilateral limb inter-coordination. Furthermore, the patient active participant assessment is integrated into the system for real-time rehabilitation training evaluation by an sEMG-based elbow joint force estimation method. Based on the experimental results, two main aspects are discussed in this section, including the sEMG-based elbow joint force estimation and the MVF-based human–robot interface for the bilateral neuro-rehabilitation robot system.

### 6.1. Comparison of the sEMG-Based Active Force Estimation with the State-of-Art

To realize the EMG-based force estimation, Zhang et al. [15] used the sEMG signals from the four muscles of the forearm to predict the muscle strength of the wrist joint. The sEMG signals were processed and calculated to obtain the muscle activity during the downward touch motion. Then the obtained muscle activity was used as the input of an artificial neural network (ANN) classifier to recognize the various motions. A developed prediction function was integrated into the muscular model for force prediction. As there were some discontinuous points in the prediction results, a smooth algorithm was utilized to obtain the final predicted force results. The total performance of this prediction method can reach that the average correlation coefficient $R^{2}\;$ is 0.9085. However, as the parameters of the muscular model function are complex and time-consuming, this method may be inconvenient for individuals. Hajian et al. [37] studied the generalized EMG-based isometric contact force estimation method using a deep convolutional neural network. The HD-sEMG signals from the three elbow flexor muscles were collected by 21 channels. The total 16 kinds of sEMG features from the time-domain and frequency-domain were calculated as the input of the proposed CNN-FLF model. This model can reach a high estimation performance for the NMSE to be 1.60 ± 3.69. However, the multi-feature input vector and deep CNN model requires high computing power to ensure real-time estimation performance. Zhang et al. [38] proposed a novel force estimation method using muscle activation heterogeneity analysis and kurtosis-guided filtering. In this study, a novel preprocessing method was designed for high accuracy estimation. First, the HD-sEMG signals were decomposed by principal component analysis, and then, a heterogeneity analysis was conducted. Finally, a kurtosis-guided filter was utilized to process the selected principal component to get the input signals. The model can realize the correlation coefficient $R^{2}$ from 0.877 to 0.955. Unfortunately, similar to the above two works, the time-consuming data processing may lead to the difficulty of real-time estimation. All the comparison results are summarized in Table 2.

As the sEMG-based elbow joint force estimation method was proposed for the real-time patient active assessment, the real-time performance is the crucial factor for real rehabilitation scenarios. In this study, only two channels of sEMG signals and four time-domain features were utilized, but the average $R^{2}$ is 0.9562 and the RMSE is 1.8935. Although more information can be saved by more multi-features extraction or more complex computing model, the real-time performance is decreased by more features and more complex model calculations. Too large of a feature calculation will fail to estimate the elbow joint force in real-time; the balance between the estimation accuracy and real-time estimation speed should be considered in real applications. The real-time performance of the proposed multi-feature vector has also been validated for the feature extraction computing time to be under 0.1 s, which is acceptable as it is under a single sliding window length. Therefore, the trade-off between the complex model and computational amount should be considered for achieving high accuracy estimation in real-time.

### 6.2. Analysis of the Efficiency of the MVF to the Bilateral Rehabilitation

**Figure 14:**
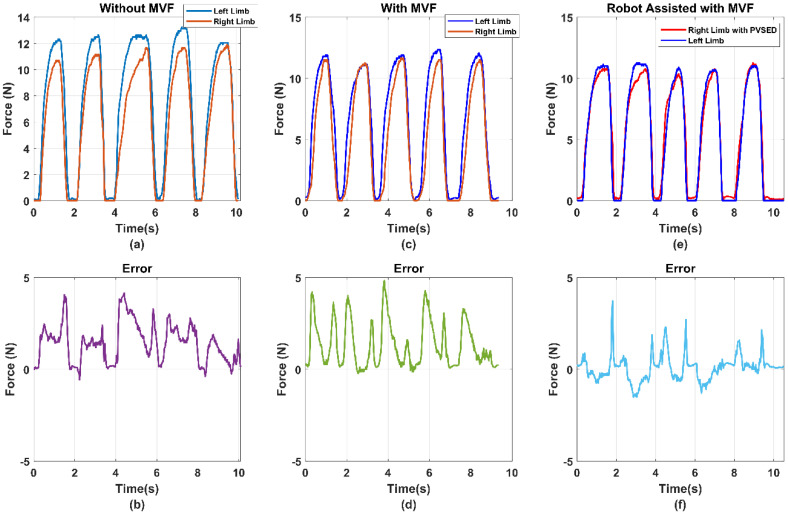
The comparison results of subject 2 in the three conditions, with/without the MVF and robotic assistance with MVF. (**a**) the bilateral isometric force task without MVF; (**b**) the errors of the bilateral isometric force task without MVF (**c**) the bilateral isometric force task with MVF (**d**) the errors of the bilateral isometric force task with MVF (**e**) the bilateral isometric force task with MVF and Robotics assistance. (**f**) the errors of the bilateral isometric force task with MVF and Robotics assistance.

**Figure 15:**
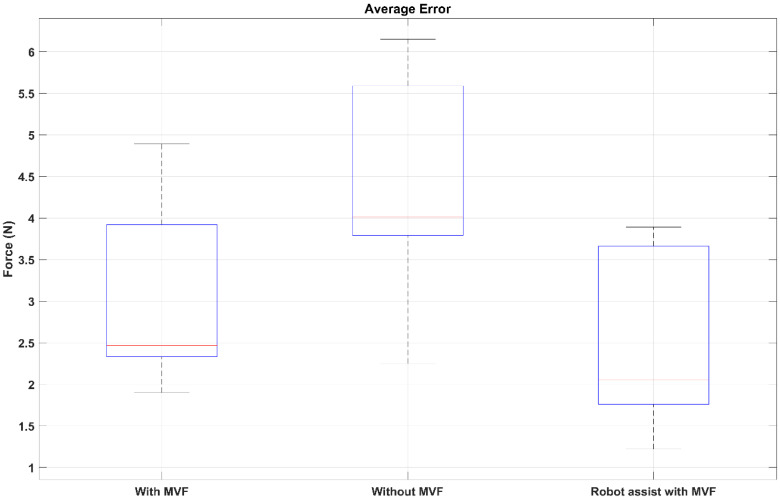
The average errors of both subject 1 and subject 2 in the three conditions, with/without the MVF and robotic assistance with MVF.

As the one-side disability of the hemiplegia patients, the inter-coordination of the bilateral limbs should be particularly considered in bilateral rehabilitation. In this study, the isometric output joint force of the bilateral limbs was selected as the rehabilitation training task to promote the inter-coordination of the bilateral limbs. In this task, the equal output force of the bilateral upper limb elbow joint was expected for better coordination. For hemiplegia patients, it is difficult to complete the equal bilateral output force of the elbow joint without any assistance or feedback. To induce the patient’s active adjustment of the bilateral limb inter-coordination, a mirror visual feedback-based human–robot interface was designed. To prove the efficiency of the MVF-based human–robot interface, a comparison experiment was conducted. The subjects were instructed to perform the bilateral isometric lifting task in three different conditions, including without MVF, with MVF, and robot-assisted with MVF. The comparison results are shown in Figure 13 and Figure 14. The average errors of the without MVF condition, with MVF condition, and the robot-assisted with MVF condition are 2.49, 4.02, and 2.04, respectively (shown as Figure 15).

From the experimental results, it is obvious that the subject performing the task with MVF has a smaller average error than the condition without MVF. As the isometric force is limited, the absolute equal bilateral output force is difficult to realize even for healthy subjects. However, it may be caused for the isometric output force task. If the training task is an isotonic lifting task, e.g., lifting a stick and keeping it horizontal, the good completion of this task can be estimated for healthy subjects. This phenomenon can also be used to explain that assistance or feedback is necessary for hemiplegia patients. When the MVF human–robot interface was provided to the subjects, the average error declined significantly. Benefiting from the MVF, the output force of bilateral limbs can be clearly obtained by the visual feedback, which means the subject can voluntarily regulate the output force of bilateral limbs for better inter-coordination. Considering the one-side disability of hemiplegia, the PVSED was utilized to assist the patients in real-time according to the different errors of bilateral output force. In the comparison experiment, the subjects were asked to perform the same task wearing the PVSED and the smallest error was obtained among three conditions. It might be caused by the control strategy of the PVSED. Due to the force error that was used as the input of the PID controller, the PVSED will be quickly activated once the error is too significant. The assistance of the PVSED will not be delivered to the subject if the force error is too small. However, the too quick regulation of assistance may lead to the high instantaneous contact force, which must be forbidden in rehabilitation scenarios. Therefore, the parameters of the PID controller were set as a relatively low value (Section 4).

## 7. Conclusions

As the mirror neurons of brains, bilateral rehabilitation training is considered a promising way to induce brain plasticity for hemiplegia patients. In this paper, a mirror bilateral neuro-rehabilitation training system with sEMG-based patient active participation assessment was proposed for the bilateral isometric force output coordination of the upper limb elbow joint. With the mirror visual feedback of the human–robot interface, the hemiplegia patients could perform bilateral isometric lifting tasks with modulated robotic assistance intuitive cognition of motor control of bilateral limbs. To realize fast and adaptive real-time active force assessment, a backpropagation neural network was utilized to map the relationship of the sEMG signals and elbow joint output force by a time-domain multi-feature vector. This active force estimation enables the therapists and patient to observe the patient’s active participation effort during the rehabilitation training for quantitative motor recovery evaluation. Considering the one side disability of the hemiplegia patients, the PVSED rehabilitation robotics was employed in this system for real-time assistance of bilateral rehabilitation. The dynamics of the PVSED were analyzed and adapted for the isometric lifting task requirements. Furthermore, a PID controller was implemented in the robotic control framework for precise and fast output force tracking.

Preliminary experiments were carried out to evaluate the feasibility of the real-time active force estimation and bilateral isometric force output assistance. As the three phases for BPNN model training, validation, and testing, the feasibility and effectiveness of the sEMG-based active force estimation method have been proven with good real-time performance. In the five experimental trials of two healthy male volunteers, the experimental results showed that the proposed mirror bilateral neuron-rehabilitation system allowed the patients to perform bilateral equal isometric output force with robotic assistance. Furthermore, a comparison experiment was conducted to validate the effect of the MVF and robot assistance on the isometric force inter-coordination of bilateral limbs. The future work will mainly focus on involving hemiplegia patients to carry out the controlled clinical trials.

## Figures and Tables

**Table 1:** Multi-feature vector selection and equations.

Feature	Equation	Description
Mean absolute value (MAV)	$\mathrm{MAV}=\frac{1}{n}\overset{n}{\underset{i=1}{\sum }}\left|x_{i}\right|$	The average of absolute value of the EMG signals amplitude in a segment
Root mean square (RMS)	$\mathrm{RMS}=\sqrt{\frac{1}{n}\overset{n}{\underset{i=1}{\sum }}x_{i}^{2}}$	The measure of power of the EMG signals which can be calculated as the amplitude modulated Gaussian random process
Difference absolute standard deviation value (DASDV)	$\mathrm{DASDV}=\sqrt{\frac{1}{n-1}\overset{n-1}{\underset{i=1}{\sum }}{\left(x_{i+1}-x_{i}\right)}^{2}}$	The standard deviation absolute value of the difference between the adjacent samples of EMG signals
Wavelength (WL)	$\mathrm{WL}=\overset{n-1}{\underset{i=1}{\sum }}\left|x_{i+1}-x_{i}\right|$	The measure of complexity of the EMG signals which defined s cumulative length of the EMG waveform over the time segment

**Table 2:** Comparison with the state of art.

Research	Joints	EMG Channels	Features	Model	Other Sensors	Results
Zhang [15]	Wrist	4	Muscle activation	ANN and Prediction Function	MTx sensor	$R^{2}$: 0.9085
Hajian [37]	Elbow	21	Temporal and Spectral information (16 in total)	Deep convolutional neural networks	no	NMSE: 1.60 ± 3.69
Zhang [38]	Elbow	128	Principal component analysis (PCA) and Heterogeneity information	Optimal Principal Component Selection and kurtosis-guided filter	no	$R^{2}:$ 0.877~0.955
This work	Elbow	2	MAV, RMS, DADSV, WL	BPNN	no	$R^{2}:$ 0.9562 RMSE: 1.8935
